# Bridging biodiversity and regenerative development: Conceptual groundwork for life-affirming relationships

**DOI:** 10.1007/s13280-026-02398-8

**Published:** 2026-04-21

**Authors:** Anu Veijalainen, Matti Salo, Henna Rouhiainen, Juulia Räikkönen, Ilari E. Sääksjärvi

**Affiliations:** 1 Department of Biodiversity Sciences, University of Turku, 20014 Turku, Finland; 2 Natural Resources Institute Finland (Luke), Kulttuurikuja 1, 20800 Turku, Finland

**Keywords:** Diversity, Planetary boundaries, Regeneration, Social-ecological systems, Sustainability, Worldviews

## Abstract

The global decline of biodiversity demands transformative change that realigns human activity with planetary processes. Regenerative development may facilitate systemic transformation but remains underexamined in relation to biodiversity. We explore the relationship between biodiversity and place regeneration, conceptually aligning biodiversity enhancement and regenerative development. This requires adopting a refocused concept of biodiversity as a dynamic, relational attribute of life that engenders biocultural diversity. Similarly, regenerative development promotes co-evolving, life-affirming relationships within and between human and natural systems. Regenerative design may catalyse regenerative development capacity in communities by engaging participants in regenerative dynamics, potentially increasing biodiversity. However, the intentional incorporation of actions explicitly aimed at enhancing biodiversity remains necessary. Challenges persist due to entrenched anthropocentric worldviews and the difficulty of including human expressions within biodiversity. The refocused biodiversity concept offers a feasible leverage point for fostering transformative change within the narrowing timeframes to halt biodiversity decline.

## Introduction

Human activities have exceeded several planetary boundaries critical for maintaining the stability and resilience of Earth’s systems, posing a significant threat to the future of humanity and countless other forms of life (IPBES 2019, 2024; PBScience 2025). Among these boundaries, the integrity of the biosphere is particularly concerning, as its status reflects the accelerating decline of biodiversity—the foundation of Earth’s life-support systems (Folke et al. 2021; Solé and Levin 2022). Reversing the decline of biodiversity and fostering sustainability requires transformative change: a systemic reorganisation that encompasses technological, economic, and social dimensions, along with the underlying paradigms, goals, and values that shape human behaviour and consequently the rest of nature (IPBES 2019, 2024).

Regeneration has become an emerging approach to driving transformative change (Fischer et al. 2024). It describes life processes that develop through positive reinforcing cycles (IPBES 2024). In our Western scientific focus, regeneration is a meta-concept whose meaning is gradually becoming clarified in relation to established sustainability concepts (Fischer et al. 2026), while framing regeneration as a paradigm or ideology remains contested (Taveras-Dalmau et al. 2025). Regenerative strategies aim to mobilise life’s inherent capacity to thrive and can thereby support biodiversity conservation (IPBES 2024).

However, regenerative strategies are vulnerable to co-optation and their relationship with biodiversity remains insufficiently explored. Regenerative agriculture can be a strategy for increasing biodiversity (Khangura et al. 2023); yet, agri-food corporations in this domain tend to focus on agricultural practices rather than socio-economic-ecological interactions, limiting its suitability to regeneration studies (Bless 2025; Gosnell and Gordon 2025; Taveras-Dalmau et al. 2025). Scientific attention on biodiversity has been scant in other domains of regeneration, such as in tourism (e.g., Bellato et al. 2024) and the built environment (e.g., Mang and Reed 2012). The lack of clarity hinders a deeper understanding of how place-based regenerative strategies could address the decline of biodiversity as part of broader efforts to stay within planetary boundaries, while also supporting human flourishing.

Addressing the increasing separation between humans and more-than-human nature, and instead, fostering mutualistic relationships among living entities, has underpinned Western regenerative practice for over three decades (e.g., Mang and Reed 2012; Buckton et al. 2023; Fischer et al. 2026). This perspective is more recent in biodiversity conservation theory (IPBES 2024; Reyers and Bennett 2025). Moreover, beyond frontline conservation, the dominant ways in which humans use space and resources—and manage biodiversity through these practices—often fail to recognise the embeddedness of humans, local cultural values, and long-term human well-being within nature (Pascual et al. 2021), leading to, for example, the insufficient inclusion of ecocentric and local viewpoints and values (Washington et al. 2024).

The relationship between biodiversity and regeneration requires clarification to better support sustainability conversations. This need echoes recent efforts to clarify the relationship between regeneration and resilience (Fischer et al. 2026). Given that contemporary interest in regeneration has been driven most prominently by the field of regenerative development and design (Reed 2007; Fischer et al. 2026), this paper explores the relationship between biodiversity and regenerative development. By conceptually aligning biodiversity enhancement and regenerative development, we aim to advance dialogue that supports their shared pursuit of life-affirming transformation. We begin by framing biodiversity as a foundational attribute of life and introducing the process of regenerative development. The paper then highlights key intersections between biodiversity and regenerative development, paying particular attention to how worldviews shape understanding. The final discussion considers how integrating these concepts and processes can inspire holistic, place-based approaches to safeguarding planetary boundaries.

## Theoretical foundations

### Biodiversity—concept, concern, and conservation

The discipline of conservation biology emerged in the 1980s with the specific mandate of preserving what became known as biological diversity (Soulé 1985), later abbreviated as biodiversity (Wilson 1988). In 1992, the convention on biological diversity (CBD) established three key objectives: conservation of biodiversity, sustainable use of its components, and equitable sharing of benefits arising from the utilisation of genetic resources. This expanded the understanding and importance of biodiversity beyond conservation biologists to a broader community of policymakers, stakeholders, and the public.

While there is no universal definition of ‘nature’ (Keune et al. 2022), biodiversity is widely understood as a multidimensional characteristic of nature, describing the variability of life forms from molecules to ecosystems (Pascual et al. 2021). The CBD defines biological diversity as “the variability among living organisms from all sources including, inter alia, terrestrial, marine, and other aquatic ecosystems and the ecological complexes of which they are part; this includes diversity within species, between species, and of ecosystems” (UNEP 1992: 3). Different systems and time periods exhibit unique levels and configurations of diversity. In essence, diversity is a relational attribute that describes the variability among living biophysical entities rather than simply referring to the collection of these entities themselves.

More specifically, biodiversity describes the variability of taxonomic (groups of organisms), structural (spatial arrangement), and functional (interactions among organisms and their abiotic environments) characteristics of biotic systems across spatial and temporal scales. In complex adaptive systems like ecosystems—characterised by interconnected components, nonlinear dynamics, and the ability to self-organise and adapt in response to environmental changes—biodiversity dynamics emerge from local biotic and abiotic interactions within a larger system (Solé and Levin 2022). As living organisms, humans are integral components of this complexity. Therefore, human social and cultural systems are also linked to the diversity of life, although direct analogies between biodiversity and socio-cultural diversity may be oversimplifications (Pretty et al. 2009; Eriksen 2021; Levis et al. 2024).

Biodiversity is generally undergoing a significant overall global decline driven by human activities (IPBES 2019). This alarming trend has prompted political commitment and intergovernmental action to halt and reverse the decline (CBD 2022). However, progress remains inadequate, and reversing the current negative trajectory requires a substantial escalation in both ambition and effectiveness (Mace et al. 2018). Goals of this magnitude cannot be achieved without the integration of biodiversity conservation into broader social and cultural frameworks that address the root causes driving the decline of biodiversity (Leadley et al. 2022)—particularly the perceived and reinforced human disconnection from nature (IPBES 2024).

Despite growing recognition of biodiversity decline and the emergence of more relational framings, biodiversity management practices still largely fall short of embracing this relational turn (Reyers and Bennett 2025). Dominant approaches include mitigating degradation or restoring ecosystems to prior states, instead of proactively enhancing biodiversity or deepening the understanding of its values (Kenter et al. 2025), or enabling “evolutionary potential for yet unknown biodiversity” (Herrmann-Pillath et al. 2023). This gap highlights the need for research that transcends current utilitarian frameworks and engages with the relational foundations of biodiversity and its co-evolving connections with people, thereby informing more holistic and forward-looking management of socio-ecological systems. This sets the stage for examining how place-based regeneration can complement biodiversity conservation and enhancement—understood as intentionally improving the state of biodiversity, with gains counting as enhancement only when they are beneficial within relevant spatial and temporal contexts.

### Regeneration—process, potential, and place-based development

#### Beyond restoration

Regeneration, as an inherent phenomenon of life, occurs across multiple scales from cellular processes to ecological succession. Regenerative socio-ecological systems foster dynamics that enable continuous life-affirming adaptation in the system’s state, resulting in higher resilience and thriving (Fischer et al. 2024, 2026). The Intergovernmental Science-Policy Platform on Biodiversity and Ecosystem Services (IPBES) refers to regeneration as processes by which socio-ecological systems revive themselves after disturbance and evolve through positive reinforcing cycles that allow for the emergence of, for example, new entanglements of biological and cultural diversity (i.e., biocultural diversity; IPBES 2024; see, Cocks 2010).

When provided with energy input that initiates regenerative momentum (Fischer et al. 2024), social-ecological systems as complex adaptive systems may transform regeneratively and express regenerative capacity as an emergent property. How humans can initiate and foster the continuous emergence of regenerative capacity in social-ecological systems is inadequately understood (Gibbons et al. 2020). Further, whether increased regenerative capacity translates into higher biodiversity has received little scientific attention beyond the domain of agriculture in a sustainability context (Khangura et al. 2023).

Shifting to regenerative processes requires a fundamental change in perspective. While restoration activities are one way for humans to initiate the revival process, restoration still suggests humans doing things *to* nature, whereas regeneration refers to humans co-evolving *with* and participating *as* nature (e.g., Du Plessis 2022; IPBES 2024). Aligning human activities with planetary processes has gained traction as a strategy for enabling life-affirming change in various societal domains critical to the state of biodiversity, such as agriculture, food systems, business, organising, tourism, and the built environment (Buckton et al. 2023). According to IPBES (2024), regenerative strategies can support cultural values, sustainable production, and biodiversity conservation.

As interest in regenerative strategies grows, diverse interpretations of what constitutes regenerative processes have emerged (e.g., Bellato and Pollock 2023). This has highlighted the need for greater coherence in regenerative discourse to prevent fragmentation, facilitate scalability, and strengthen links between regeneration and established concepts in sustainability science (Fischer et al. 2024, 2026). Critical reflection on the paradigmatic foundations of regeneration is necessary before assigning it a ‘new’ paradigm (Taveras-Dalmau et al. 2025).

At the individual level, engagement in regenerative change may follow from at least two different stances: (i) to create regenerative dynamics in a system outside of oneself, such as in agricultural soil, while intending to remain personally untouched by the process (restorative stance; Khangura et al. 2023); and (ii) to facilitate the regeneration of a social-ecological system while embedded in the system, motivated to personally co-evolve within it (regenerative stance; Taveras-Dalmau 2024; Gosnell and Gordon 2025). The related fields of regenerative development, design, sustainability, cultures, and futures represent the latter style (Mang and Reed 2012; Robinson and Cole 2015; Wahl 2016; Camrass 2020). They connect various stakeholders across sectors, aiming to harmonise human activities with the continuous evolution of life in a place-based manner, for example, within a watershed (Hes et al. 2018; Gibbons 2020; Gibbons et al. 2020; Bellato et al. 2024).

#### Regenerative development

The field of regenerative development was introduced in the mid-1990s by consultants in the built environment sector, who viewed environmental degradation as a symptom of human cultural and psychological disconnection from nature (Mang and Reed 2020). Here, the word ‘development’ refers both (i) to the progression that occurs as people engage in regenerative practice, developing the inner capacities and capabilities needed to catalyse regeneration in outer systems while drawing on multiple disciplines and epistemologies, and (ii) to social-ecological systems subsequently developing regenerative capacity and capability through mutually life-affirming, cross-scale interactions (e.g., Mang and Reed 2012), which correspond to regenerative dynamics (Fischer et al. 2024).

Although the field has been described in scholarly literature, it remains primarily popular among practitioners (e.g., Gorissen et al. 2024). The limited scientific research on regenerative development leaves its relevance and assessment uncertain, pushing scholars towards grey literature and non-academic training, while the lack of established metrics that would reflect its dynamic, relational, and evolving nature further hampers assessment (Gibbons et al. 2020; Graves 2020; Cianchi et al. 2023; Toner et al. 2023).

In theory, regenerative development promotes human alignment with planetary processes. The living systems perspective is central to the field. Living systems are complex adaptive systems that integrate human and natural systems, organise and co-evolve as interdependent nested wholes, and emphasise the system’s capacity to actualise higher potential (Du Plessis 2008; Mang and Reed 2012). In practice, regenerative development shifts the focus from managing system components to fostering the patterns of relationships between components that are supportive of resilience, self-organisation, and the production of life-affirming emergent outcomes. In contrast, many discourses of regeneration do not fully incorporate this living systems approach, including some within regenerative agriculture (Hes and Rose 2019; Gosnell and Gordon 2025) and tourism (Bellato and Pollock 2023). The living systems perspective also distinguishes regenerative development from other holistic and interdisciplinary approaches to safeguarding social–ecological health, such as Planetary Health (Lerner and Berg 2017).

Regenerative development emphasises the unique human capacity to intentionally create conditions that support the thriving of socio-ecological systems, akin to how keystone species function (e.g., Gorissen 2024; Gorissen et al. 2024), and which resilience thinking calls ‘biosphere stewardship’ (Fischer et al. 2026). Regenerative development aims to enable the unfolding of life’s evolutionary potential in context-specific ways by focusing on the quality of social-ecological relationships and engaging local communities (Reed 2007; Hes et al. 2018; Gibbons 2020; Du Plessis 2022; Toner et al. 2025). Rather than pursuing predetermined goals or applying generic solutions, regenerative development seeks to continually enliven systems by reinforcing the unique characteristics of people, communities, and ecosystems through feedback loops. This approach surpasses the conventional aims of sustainability and can boost resilience thinking (Gibbons 2020; Fischer et al. 2026). Human activity becomes value-adding when it increases the living system’s capacity to sustain and evolve higher orders of vitality and viability for the life of a place (Mang and Reed 2014).

Regenerative development reflects a departure from the prevailing perspective on life, which is characterised by linearity, reductionism, and dualism, positioning humans outside or above natural systems; instead, the living systems perspective of regenerative development highlights non-linearity, interdependence, emergence, and co-evolution (Benne and Mang 2015; Du Plessis and Brandon 2015). This repositions humans as participants within the living system, fostering a sense of belonging and aligning human actions with the planet’s regenerative capacity. In the field of regenerative development, these perspectives are typically referred to as the mechanistic worldview and the ecological or living systems worldview, respectively (e.g., Du Plessis and Brandon 2015). While philosophically unclear concepts, the former reflects anthropocentric and the latter ecocentric worldviews. Among ecologically focused worldviews in general, the living systems perspective is distinctive in highlighting the connection between human mental phenomena and the quality of relating in shaping systemic outcomes (e.g., Du Plessis 2008; Du Plessis and Brandon 2015)—an epistemology rarely addressed in systems transformation work in modern societies (Scharmer and Pomeroy 2024).

Together, regenerative development and its complementary approach, regenerative design, seek to co-create systems that enhance the healthy interdependence of social and ecological communities, enabling these systems to express their potential for diversity, complexity, and creativity (Mang and Reed 2012). Sustainable development projects typically pursue narrower goals (Hes et al. 2018). In a place-based geographic context, the design process begins by exploring the unique characteristics of the place; it continues by envisioning how local projects can participate in their shared living system in ways that leverage its overall life-affirming potential (Mang and Reed 2020). The process involves integrating information on the natural and cultural history, choosing suitable frameworks, and applying diverse ways of knowing (e.g., Mang and Reed 2012; Toner et al. 2023). While regenerative development and design can deliver material outcomes, the practitioner’s main goal is to apply and build capacity and capability into the living system—they empower the local community to continue the co-evolutionary process beyond the design project’s scope (e.g., Gibbons et al. 2020). This may manifest just as subtle shifts in the sense of caring within and between people and their shared place.

Original research applying regenerative development and design theory remains scarce. However, a growing number of case studies help clarify how regenerative projects are implemented and what impact they generate. These studies highlight a departure from traditional sustainability efforts, which often rely on reductionist methodologies and pursue predetermined goals through a linear progression. The research describes community engagement processes that involve a wide range of experts and stakeholders, including marginalised local voices, and follows a dialogic, iterative approach that adapts to feedback with the aim of cultivating care and commitment to a place-based vision (Hes et al. 2018; Slawinski et al. 2021; Toner et al. 2023, 2025). In contrast, many conventional biodiversity and sustainability initiatives tend to be expert-driven, with limited and cautious local involvement, rigid goals that hinder adaptive learning, and little emphasis on inclusive dialogue, trust-building, or long-term community ownership after project completion (Kiss et al. 2022; Herrmann-Pillath et al. 2023).

In a rare study comparing ‘business-as-usual’ and regenerative development plans for the same site, researchers revealed a transition from generalised procedures, problem-solving, and socio-economic priorities towards place-sourced planning and the enhancement of socio-ecological systems from a living systems perspective (Hes et al. 2018). Although many of the regenerative plan’s actions still resembled restorative interventions, the comparison highlighted a key distinction. The conventional approach aimed for a relatively static solution of creating wildlife refuges, while the regenerative plan sought to create conditions conducive to the ongoing development of resilient ecosystems, for example, by investing in gradually improving soil conditions throughout the site.

Despite the field’s potential for fostering transformation, its connections with biodiversity are scientifically underexamined (Cianchi et al. 2023). While some non-academic publications underline the importance of high biodiversity for socio-ecological resilience (Gorissen 2024), references to biodiversity, including attributes like species richness or ecological recovery, are absent or ambiguous in scholarly publications (e.g., Gibbons et al. 2020). Some case studies have highlighted the role regenerative design can play in increasing biodiversity and reported success through the return of certain species (Mang and Reed 2012; Hes and Bush 2020), thereby miscommunicating the fact that biodiversity only manifests in varying degrees rather than as the presence or absence of components. However, regenerative development may improve biodiversity indirectly, for example, through promoting local food systems or reducing reliance on extractive practices (Hes and Rose 2019; Bellato et al. 2024).

The limited attention suggests that many authors have equated biodiversity with its biophysical components, rather than viewing diversity as variability—and therefore as a dynamic, relational attribute of life that emerges from nature’s dynamics, including biocultural transformation. While some studies with a broad scope on regenerative systems have correctly approached biodiversity as a descriptor of variability and recognised diversity as essential to fostering regenerative dynamics, they also remain unclear about how biodiversity relates to similar concepts like biocultural diversity (Buckton et al. 2023). Overall, scientific literature does not sufficiently explain biodiversity as both a prerequisite and an outcome of regeneration.

## Aligning biodiversity and regenerative development

Table 1 presents proposed framings and aligns key concepts relevant to bridging biodiversity and regenerative development to support sustainability conversations. When understood in the above-described way, biodiversity can be viewed as a characteristic of socio-ecosystemic transformation process, as a concept that captures the changing variety of biotic entities, their spatial arrangement, and functional expressions. Supporting the natural potential for biodiversity can be considered a quality of this transformation. In a similar vein, regeneration is a life-affirming transformation process. The aspiration of regenerative development is to build capacity in living systems, so that communities will engage in fostering the enhancement of socio-biocultural complexity. Aligning the processes of regenerative development and biodiversity enhancement requires approaching biodiversity as variability rather than components and adopting a living systems perspective. Other considerations also need to be addressed to bridge biodiversity and regenerative development (Table 2).

**Table 1:** Proposed alignment of relevant terms to support conversations bridging biodiversity and regenerative development. ^a^Biodiversity enhancement is the intentional improvement of the state of biodiversity—recognising that gains count as enhancement only when they are beneficial within the relevant spatial and temporal context

Complex adaptive system	Place-specific socio-ecological system	
Process	Biodiversity enhancement^a^	Regenerative development
Transformation	Socio-ecosystemic functioning	Regeneration
Quality of transformation	Potential-enabling	Life-affirming
Emergent property	Biodiversity	Regenerative capacity
Aspiration	Improved state of biodiversity	Higher capacity to foster socio-biocultural complexity
Required view	Refocused concept: biodiversity as the variety of biotic entities, their spatial arrangement, and functional expressions, is linked to biocultural variability	Living systems perspective

**Table 2:** Suggested views on key concepts to enable bridging biodiversity and regenerative development, contrasted with narrower perspectives that may hinder alignment and conversations

Key concept	Narrow view	Prerequisite for alignment
Diversity	Pool of components	Emergent feature of relational systems
Biodiversity	Pool of genes, species, and ecosystems	Dynamic, relational attribute of life that engenders biocultural diversity
Ecological complexity and organismal diversity	Obstacles to productivity	Opportunities to enhance resilience and functionality within human systems
Respect for the more-than-human nature	Valued for its benefits to humans	Valued for its relational value beyond the benefits or harm it engenders to humans

Worldviews, as deeply rooted cultural, social, and philosophical frameworks, refer to how individuals and societies interpret the world. They affect what is considered valuable and worthy of protection, shaping how biodiversity is understood and engaged. The field of regenerative development often emphasises worldviews and approaches them as mechanistic or living systems—while advocating for the latter. In this context, a mechanistic worldview tends to be dominated by interpretations of the reality as something that can be strategically manipulated based on the idea that “the universe as a whole and all of its material components, including living organisms, function like mechanical systems that are governed by universal laws” (Benne and Mang 2015: 43). A living systems worldview, on the other hand, “does not negate or replace the mechanistic paradigm, but adds to the knowledge base by providing a different perspective which reveals different types of knowledge” (Du Plessis and Brandon 2015: 55), and tends to emphasise understanding the universe holistically as “an interdependent and interconnected web of multileveled structures of complex living systems” (Benne and Mang 2015: 44) that is too complex to be mechanistically manipulated.

A distinction between mechanistic and ecologically informed living systems worldviews is not necessarily possible or needed from the perspective of biodiversity (Peterson 2021). Nevertheless, shifting emphasis from a worldview dominated mostly by mechanistic understandings of life towards an ecologically focused perspective, where humans are regarded as embedded within nature, can fundamentally reshape biodiversity conservation and enhancement efforts. Such a shift highlights the role and value of biodiversity as a property of nature’s systems on which humans depend. An ecologically focused perspective emphasises biodiversity’s dynamic, relational, and evolving nature, framing it as a continuum of interactions within and between nested living biophysical entities, rather than only as the existing pool of genes, species, and ecosystems. Understanding biodiversity through this lens refocuses on how humans also have the capacity to enhance biocultural diversity within socio-ecological systems. This shift of understanding offers transformative pathways to interdependent futures that intersect with regenerative development.

## Discussion

Here, we have explored how regenerative development, arising from ecologically focused worldviews, can inform biodiversity conservation and enhancement efforts, and vice versa, to accelerate sustainability. This comparison revealed substantial common ground between the two approaches, suggesting that their conceptual alignment can foster more integrated pathways, even as challenges remain.

IPBES (2024) asserts that addressing biodiversity decline and achieving transformative change depends not only on *what* actions are taken, but also on *how* they are enacted, including the principles, values, and practices that shape them. According to Taveras-Dalmau et al. (2025), advocates of regeneration may be merely advancing an ideology, as they are prone to a phenomenon called paradigm blindness if they fail to recognise or value alternative perspectives. The authors also suggest that many individuals engaged in systems change have long focused on biodiversity conservation and local well-being, and that adopting regeneration as an alternative paradigm may result in only nominal changes to their existing practices. This concern aligns with broader challenges of premature and misleading co-optation of regeneration.

At the same time, regenerative development offers guidance on how transformative change might be embodied and enacted. Its emphasis on plural and place-based epistemologies can reveal biodiversity-related values that are often overlooked in conventional biodiversity approaches. The living systems perspective connects biodiversity efforts with life principles and personal transformation, potentially shifting worldviews.

Nevertheless, communication around worldviews in regenerative development remains relatively limited and largely systems-focused. The field often continues to prioritise anthropocentric ways of knowing and rely on frameworks that echo mechanistic approaches to managing life. Some critics have argued that regenerative development and design have not fully abandoned the mechanistic worldview due to their ongoing reliance on ecology; instead, they have advocated for regenerative change informed by constructivist social theory, suggesting that multiple pathways to regeneration may exist (Robinson and Cole 2015; Camrass 2020).

A key limitation of mainstream biodiversity efforts is their persistence within worldviews that often position biodiversity as external to humans—a pool of items to be managed or restored. Given the rapid progression of biodiversity decline, relying on a widespread shift in worldviews to drive transformative action seems unrealistic in the short term. Changes towards relational, ecocentric, or pluricentric worldviews (Kenter et al. 2025) are typically slow and contested. Thus, we propose refocusing the concept of biodiversity as a dynamic and relational attribute of life, rather than solely as an inventory of biotic components. This refocusing may offer a more immediate and practical leverage point for nature-related planning and management to bridge entrenched human-nature divides.

Importantly, regenerative development alone does not guarantee positive ecological outcomes. While regenerative systems are often associated with increased place-based vitality and viability (Mang and Reed 2014; Gibbons 2020; Fischer et al. 2026), such unspecified changes do not necessarily translate into higher biodiversity. In theory, design for regenerative development can be a strategy for increasing biodiversity, as it can provide the initial energy input to initiate regenerative momentum and dynamics in a living system. In practice, however, strategies explicitly aimed at enhancing biodiversity are required alongside regenerative development—such as promoting protected areas and addressing telecoupling—to ensure positive biodiversity impacts.

Established approaches to evaluate regenerative social-ecological transformation or capacity are not yet available (Gibbons et al. 2020; Buckton et al. 2025). The current biodiversity and ecosystem approaches are inadequate for assessing the regenerative principle of fostering co-evolution between socio-cultural and ecological systems; addressing this gap in future indicators would encourage a broader reflection on humanity’s role within socio-ecological systems, a shift from product-oriented to process-oriented tools, and the integration of ecologically focused worldviews (Cianchi et al. 2023). Thus, refocusing the concept of biodiversity to describe a dynamic attribute of transformation rather than components, is also crucial for advancing process-focused tools that assess place-based regeneration for sustainability.

Bringing biodiversity and regenerative development into dialogue raises a critical question: does increasing biodiversity also entail enhancing the unique, potential-affirming cultural and social characteristics of geographical places, alongside their biological diversity? If so, biodiversity would align even more closely with the aims of regenerative development, which emphasise the co-evolution of human and natural systems towards realising the unique potential of place. Further research is needed to clarify the extent to which the cultural and social diversity of human systems relates to biodiversity (Otamendi-Urroz et al. 2025).

Although reductionist scientific inquiry may not inherently promote regenerative dynamics, science nevertheless has the potential to establish a shared yet context-adjustable language for stakeholders engaged in sustainability transformations. By undergoing more rigorous scientific examination and engaging actively in mainstream scholarly discussions on biodiversity, regenerative development and design could help advance further conversations and clarify their relevance. Scientific knowledge—such as multispecies approaches and insights into the cultural and philosophical foundations of worldviews and relational values—could facilitate deeper reflection on diverse perspectives within the field and support a move away from anthropocentrism. Learning the science can serve as an entry point to promoting the art of regenerative development.

## Data Availability

No data were deposited for this article, as the perspective is based on conceptual discussion and literature review.
